# Monoclonal gammopathy of renal significance (MGRS) increases the risk for progression to multiple myeloma: an observational study of 2935 MGUS patients

**DOI:** 10.18632/oncotarget.23412

**Published:** 2017-12-18

**Authors:** Normann Steiner, Georg Göbel, Patricia Suchecki, Wolfgang Prokop, Hannes Neuwirt, Eberhard Gunsilius

**Affiliations:** ^1^ Department of Internal Medicine V (Haematology and Medical Oncology), Medical University of Innsbruck, A-6020 Innsbruck, Austria; ^2^ Department of Internal Medicine IV Nephrology and Hypertension, Medical University of Innsbruck, A-6020 Innsbruck, Austria; ^3^ Department of Medical Statistics, Informatics and Health Economics, Medical University of Innsbruck, A-6020 Innsbruck, Austria; ^4^ Central Institute for Medical and Chemical Laboratory Diagnosis, Innsbruck Medical University Hospital, Medical University of Innsbruck, A-6020 Innsbruck, Austria

**Keywords:** monoclonal gammopathy of undetermined significance, MGUS, monoclonal gammopathy of renal significance, MGRS, multiple myeloma

## Abstract

**Purpose:**

Monoclonal gammopathy of undetermined significance (MGUS) is a premalignancy preceding multiple myeloma (MM) or related disorders. In MGUS, renal impairment caused by deposition of the monoclonal immunoglobulins or free light-chains monoclonal gammopathy of renal significance (MGRS) is often associated with high morbidity and mortality. We analysed the prevalence of renal impairment, clinical features and the long-term outcome in 2935 patients with MGUS.

**Methods:**

Between 1/2000 and 8/2016, 2935 adult patients with MGUS were identified in our database.

**Results:**

In 44/2935 (1.5%) patients MGRS was diagnosed. In MGRS patients, significantly more progressions to MM were observed than in MGUS patients (18% vs. 3%; P<0.001). MGRS patients showed a higher risk for progression (HR 3.3 [1.5-7.4]) in the Cox model. Median time to progression was 23 years for MGUS and 18.8 years for MGRS patients. Corresponding progression rate was 8.8 [7.2-10.7] per 1000 patient-years (py) for MGUS patients and 30.6 [15.3–61] for the MGRS group. Risk for progression within the first year after diagnosis was 1% [0.6-1.4] in the MGUS group and 10% [4-29] among MGRS patients.

**Conclusion:**

The significantly higher risk for progression to MM means MGRS patients should be monitored carefully and treated in a specialized centre.

## INTRODUCTION

Monoclonal gammopathy of undetermined significance (MGUS) is characterized by the presence of monoclonal protein in the serum of <3g/dl and a bone marrow involvement of <10% by clonal plasma cells [1]. The disease is characterized by the absence of end organ damage related to the proliferation of monoclonal plasma cells [2]. MGUS is known to be the premalignant precursor of myeloma, with a progression rate to multiple myeloma (MM) or a related malignant condition of 0.5% - 1.5% percent per year [3]. The main risk factors for progression are a non-IgG isotype, an M protein concentration of at least 15 g/l and an abnormal serum free light-chain ratio [2]. One of the defining criteria of MM is renal impairment and a serum creatinine > 2.0 mg/dl related to the plasma cell dyscrasia [4-6]. MM is known to be the most common monoclonal gammopathy to damage the kidney. Several diseases such as cast nephropathy, AL amyloidosis, monoclonal immunoglobulin deposition disease of the Randall type (MIDD), and light-chain proximal tubulopathy can result from MM [7]. Nevertheless, an increasing number of kidney diseases associated with monoclonal gammopathy do not meet the criteria for MM [4]. Patients are often diagnosed with MGUS, although they have a disease that is actually not of undetermined significance. To further explore this phenomenon, a new term was introduced: monoclonal gammopathy of renal significance (MGRS) [8]. The term was developed, firstly, to increase awareness for patients with renal impairment related to monoclonal gammopathy and, secondly, to offer them a good option for finding an appropriate therapy. Recent years have seen the proposal of a way to distinguish between MGUS and monoclonal gammopathy associated with some renal lesion. Recent studies reveal that the deposition of monoclonal protein can cause direct or indirect kidney disease [9]. The combination of MGRS patients erroneously diagnosed and the fact that treatment of MGUS is not recommended until progression to MM means patients were under-treated [10]. The lacking treatment is not important from a tumour viewpoint. However, it is crucial to protect renal function by reducing toxic monoclonal proteins. MGRS can evolve to a wide range of renal lesions and may occur only with isolated proteinuria, but also as end-stage kidney disease requiring kidney transplantation. There are two categories of MGRS disease: MGRS with organized deposits and MGRS with non-organized deposits [11]. MGRS with organized deposits includes diseases like Ig-related amyloidosis, immunotactoid glomerulopathy and type 1 cryoglobulinaemic glomerulonephritis. MGRS with non-organized lesions contain light-chain proximal tubulopathy, crystal storing histiocytosis, proliferative glomerulonephritis or C3 glomerulopathy.

The aim of our large observational study was to describe the prevalence of kidney disease in MGUS patients and to compare clinical features and risk factors for disease progression in MGUS patients with and without kidney disease.

## RESULTS

### Patient characteristics

2891 (98.5%) had MGUS without renal impairment and 44 (1.5%) showed MGRS. Table 1 summarizes demographic data and laboratory features of MGUS and MGRS patients. Concerning laboratory findings, significantly more MGRS patients had elevated urea levels (n=21, 48% vs. n=592, 21% in MGUS; P<0.001). More MGRS patients had renal dysfunction (creatinine >3 mg/dl n=7, 16% vs. n=91, 3% in MGUS; p>0.001) and elevated phosphate levels (n=7, 17% vs. n=155, 7% in MGUS; p>0.02). In the total study population 20 (46%) patients with IgG MGUS, 11 (25%) patients with IgM MGUS, three (7%) patients with IgA MGUS, one (2%) patient with IgD MGUS, and nine (21%) patients with light-chain only MGUS showed monoclonal gammopathy of renal significance (MGRS). Median LDH levels at diagnosis did not differ significantly (203 U/l [170–255] vs. 212 U/l [180–277], p=0.3]. Monoclonal protein levels differed significantly between MGUS and MGRS patients (0.3 g/dl [0.2-0.6] vs. 0.5 g/dl [0.3-1.0], p=0.02].

**Table 1:** Demographic data and laboratory features at diagnosis; comparison of the two cohorts MGUS and MGRS

Parameter	MGUS		MGRS		P
	N=2891	%	N=44	%	
Median age (range), years	68(57-76)		65(56-73)		0.3
Sex f/m					0.4
f	1247	43	16	36	
m	1644	57	28	64	
Ig heavy chain(serum)^*^					< 0.001
IgG	1946	67	20	46	
IgM	528	18	11	25	
IgA	234	8	3	7	
IgD	0	0	1	2	
biclonal gammopathy					
IgG+IgM	101	4	0	0	n.a.
IgG+IgA	39	1	0	0	
IgA+IgM	5	0.2	0	0	
IgA+IgG+IgM	2	0.1	0	0	
Light chain only	36	1	9	21	
Ig light chain(serum)^*^					< 0.001
Kappa	1644	57	23	52	
Lambda	1066	37	20	45	
Both	166	6	0	0	
Not measurable	15	0.5	1	2	
Total protein >UNV(8 g/dl)	88	8	0	0	
LDH >UNV	719	26	16	37	
Creatinine^*^					< 0.001
<1.5 mg/dl	2414	86	25	57	
1.5 - <2 mg/dl	185	7	7	16	
2 - <3 mg/dl	129	5	5	11	
≥3 mg/dl	91	3	7	16	
Urea >UNV^*^	592	21	21	48	< 0.001
Serum calcium >UNV	207	8	3	7	0.9
Phosphate >UNV^*^	155	7	7	17	0.02
Haemoglobin ≤12 g/dl	1054	37	15	34	0.7
Platelets <150,000/mm^3^	561	20	7	16	0.5
CRP >UNV	1622	58	21	49	0.2
Iron <UNV	311	14	1	3	0.04
Ferritin <UNV	176	8	1	2	0.2
Transferrin >UNV	34	2	0	0	0.4.

MGUS; MGRS (monoclonal gammopathy of renal significance); N, number of patients; Ig, immunoglobulin; UNV, upper normal value; LDH, lactate dehydrogenase.

^*^P values indicate the results of exact CHI^2^ tests or the Mann-Whitney U test (age). A P value <0.05 was considered statistically significant. No correction for multiple testing was applied.

In the MGRS group 12 (27%) patients had AL amyloidosis, nine (21%) patients a light-chain deposition disease, seven (16%) patients showed chronic kidney disease (CKD G4/G5) secondary to paraproteinemia, eight (18%) patients membranoproliferative GP, four (9%) patients membranous GP, one (2%) patient presented with mesangioproliferative GP, and three (7%) patients suffered from nephrotic syndrome. Table 2 and Table 3 specify clinical symptoms and creatinine course, association between immunoglobulin isotypes and treatment modalities in MGRS patients. All patients with AL-amyloidosis, membranoproliferative-, membranous- and mesangioproliferative glomerulopathy have been diagnosed by utilizing a renal or – in case of AL-amyloidosis– non-renal solid organ biopsy. Seven of nine (78%) of patients with light chain deposition disease were biopsy-proven, whereas two (22%) where diagnosed by massive free light chain proteinuria and increased serum-creatinine. Three patients with otherwise unexplained nephrotic syndrome and seven patients with otherwise unexplained impaired kidney function and proteinuria where also diagnosed as MGRS (see also “Patients and Methods”).

**Table 2:** Clinical characteristics of patients with MGUS associated nephropathy (MGRS)

MGRS N= 44 (100%)	
N (%)	44 (100)
Sex f/m	
f (%)	16 (36)
m (%)	28 (64)
Ig isotype	
IgG	20 (46)
IgM	11 (25)
IgA	3 (7)
IgD	1 (2)
Light chain only	9 (21)
Ig light chain	
Kappa	23 (52)
Lambda	20 (46)
Not measurable	1 (2)
Median / Mean Creatinine at baseline(range)	1,4 / 2,0 (0,9 – 2,1)
Median / Mean Creatinine at disease progression(range)	2,4 / 4,5 (1,5 – 6,0)
Pathologic urine sediment	
Yes	28 (64)
No	9 (21)
Missing	7 (16)
Renal biopsy	
Yes	27 (61)
No	17 (39)
Other organ biopsy	
Yes	5 (11)
No	12 (27)
Diagnosis of MGRS [% biopsy proven]	
AL-amyloidosis	12 (27) [100]
Light chain deposition disease	9 (21) [78]
CKD G4/5 secondary to paraproteinemia	7 (16) [0]
Membranoproliferative glomerulopathy	8 (18) [100]
Membranous glomerulopathy	4 (9) [100]
Mesangioproliferative glomerulopathy	1 (2) [100]
Nephrotic syndrome	3 (7) [0]

GN, Glomerulonephritis; CKD, chronic kidney disease; Ig, Immunoglobulin; IVIG, Intravenous immunoglobulin; NTX, kidney transplantation; IMiDs, Immunomodulatory drugs; ASCT, autologous stem cell transplant; COP, cyclophosphamide, vincristine, and prednisone; CHOP, cyclophosphamide, doxorubicin, vincristine, and prednisone.

**Table 3:** Treatment of patients with MGUS associated nephropathy (MGRS)

MGRS	N	%
**Light chain deposition disease**	9	100
No therapy	5	55.6
NTX	1	11.1
Erythropoietin	1	11.1
Mephalan/Dexamethasone + NTX	1	11.1
Hemodialysis + Bortezomib/Dexamethasone + Bortezomib/Adriamycin/Dexamethasone + ASCT	1	11.1
**AL-amyloidosis**	12	100
No therapy	2	16.7
Bortezomib	1	8.3
Bortezomib/Dexamethasone + ASCT	1	8.3
Bortezomib/Cyclophosphamide/Dexamethasone + ASCT	1	8.3
Bortezomib/Dexamethasone	2	16.7
Cladribine/Bendamustin + Rituximab	1	8.3
Cyclophosphamide/Dexamethasone	1	8.3
Hemodialysis + Bortezomib/Dexamethasone + Bortezomib/Thalidomide/Dexamethasone	1	8.3
Hemodialysis + Bortezomib/Dexamethasone	1	8.3
Bortezomib/Melphalan/Prednisone + Lenalidomide	1	8.3
**Membranous glomerulopathy**	4	100
No therapy	2	50
Erythropoietin	1	25
Cyclophosphamide/Vincristine/Prednisone + Hemodialysis + CHOP	1	25
**Mesangioproliferative glomerulopathy**	1	100
No therapy	1	100
**Membranproliferative glomerulopathy**	8	100
No therapy	2	25
Dexamethasone	2	25
Bortezomib	1	12.5
Dexamethasone + Erythropoietin	1	12.5
Bortezomib/Cladribine + NTX + Rituximab	1	12.5
Cyclophosphamide/Dexamethasone + Mycophenolate-mofetil + Hemodialysis + NTX	1	12.5
**CKD G4/5 secondary to paraproteinemia/nephrotic syndrome**	10	100
No therapy	7	70
Bortezomid/Thalidomide/Dexamethasone	1	10
Erythropoietin	1	10
Hemodialysis	1	10

N, number; NTX, kidney transplantation; ASCT, autologous stem cell transplant; CHOP, Cyclophosphamide/Doxorubicin/Vincristine/Prednisone CKD, chronic kidney disease.

### Clinical outcome in MGUS and MGRS patients

**Figure 1:**
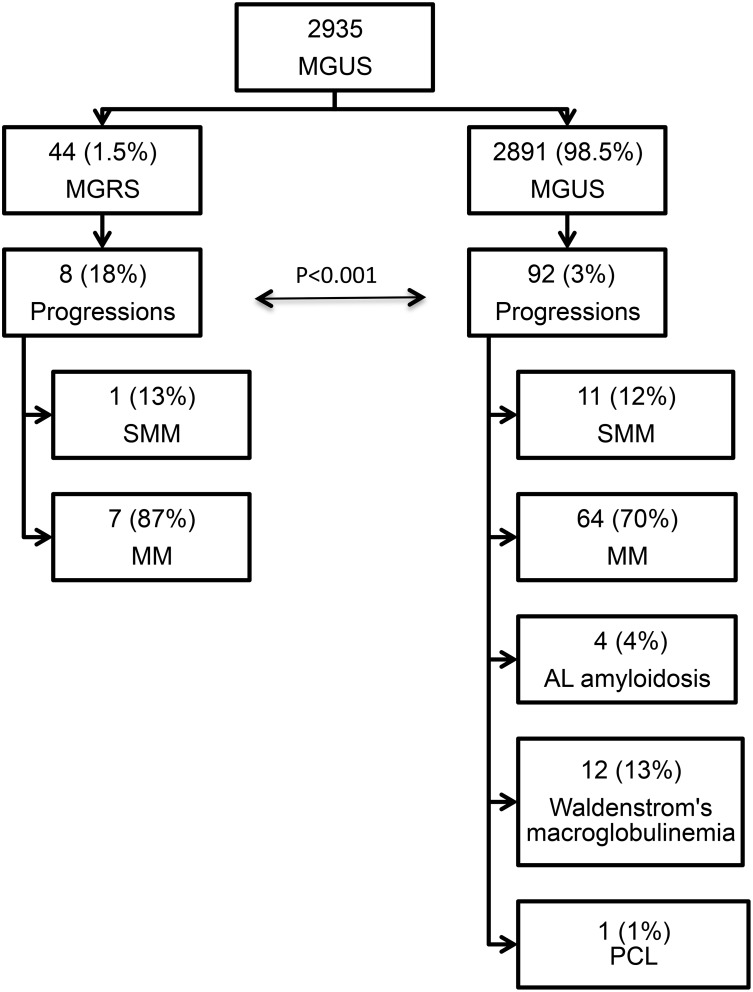
Study population overview MGUS, monoclonal gammopathy of undetermined significance; MGRS, monoclonal gammopathy of renal significance; SMM, smoldering multiple myeloma; MM, multiple myeloma; PCL, plasma cell leukemia.

Figure 1 shows an overview of the study population with regard to progression to MM or other lymphoproliferative disorders. A total of 100 (21%) patients progressed after a median time of 23 years for MGUS and 18.8 years for MGRS patients. In the MGUS cohort 92 patients progressed, 11 of whom to smoldering multiple myeloma (SMM; 12%), 64 (70%) to MM, four (4%) to AL amyloidosis, 12 (13%) to Waldenstrom's macroglobulinemia, and one (1%) to plasma cell leukaemia (PCL). In the MGRS cohort a total of eight patients progressed, 87% of whom to MM (n=7), and 13% to SMM (n=1). Significantly more progression to MM was observed in MGRS patients than in MGUS patients (18% vs. 3%; P<0.001).

Doubling of creatinine levels, renal replacement therapy, and stable renal function were defined renal endpoints and were analysed in MGRS patients. In 43 of the total 44 patients follow-up data were available. Twelve (28%) patients had stable renal function (no creatinine doubling or end-stage renal disease), creatinine levels at baseline in median were 1.4 mg/dl (SD 0.75) and at follow-up 1.37mg/dl (SD 0.91, P=0.81). In 31 (72%) patients renal disease was seen to progress; 19 (44%) of these patients showed a doubling of creatinine levels (median 1.02 mg/dl at baseline and 2.78 mg/dl at follow-up; P<0.001) and 12 (28%) patients required renal replacement therapy.

### Survival and time to progression (TTP) in MGUS and MGRS patients

**Figure 2:**
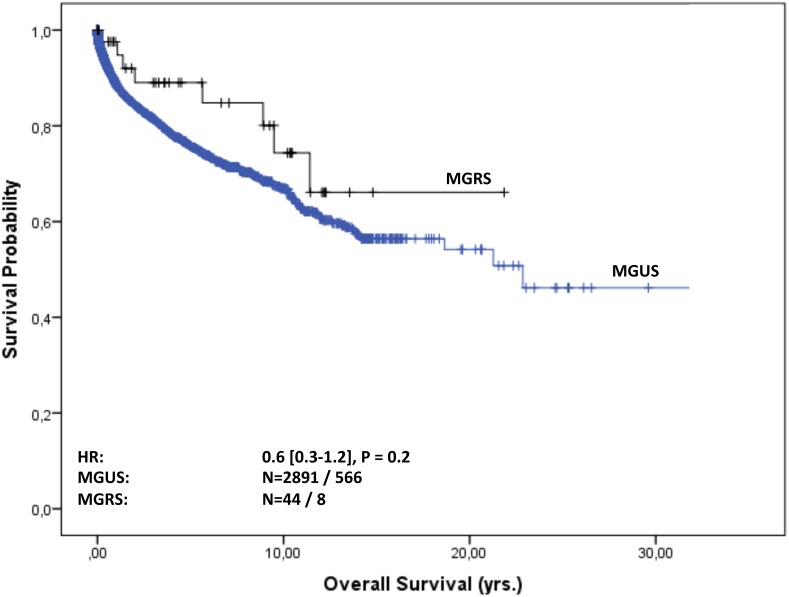
Overall survival of MGUS vs. MGRS patients Overall survival in years from MGUS diagnosis stratified by MGUS / MGRS diagnosis. The hazard ratio (HR, 95% CI) was calculated with a Cox regression model adjusted for sex, age and serum-creatinine level at baseline.

**Figure 3:**
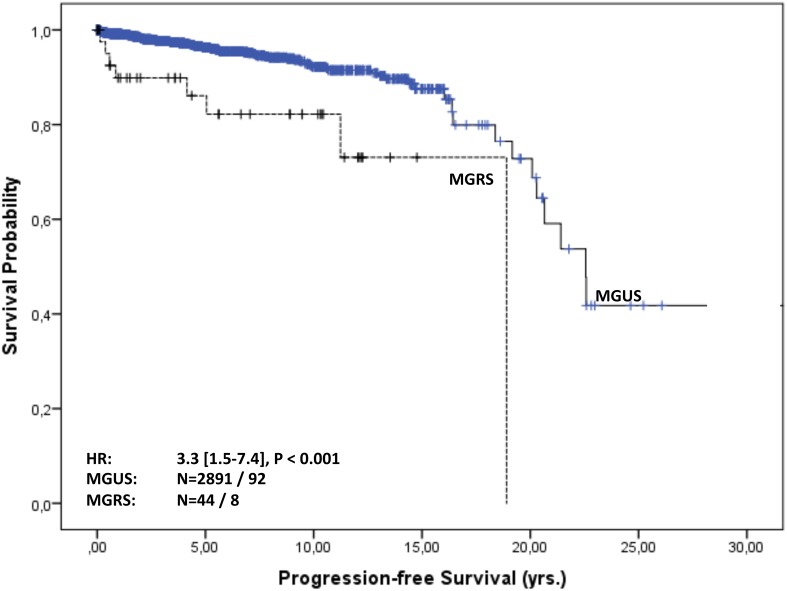
Progression-free survival of MGUS vs. MGRS patients Progression-free survival in years from MGUS diagnosis stratified by MGUS / MGRS diagnosis. The hazard ratio (HR, 95% CI) was calculated with a Cox regression model adjusted for sex, age and serum creatinine level at baseline.

**Figure 4:**
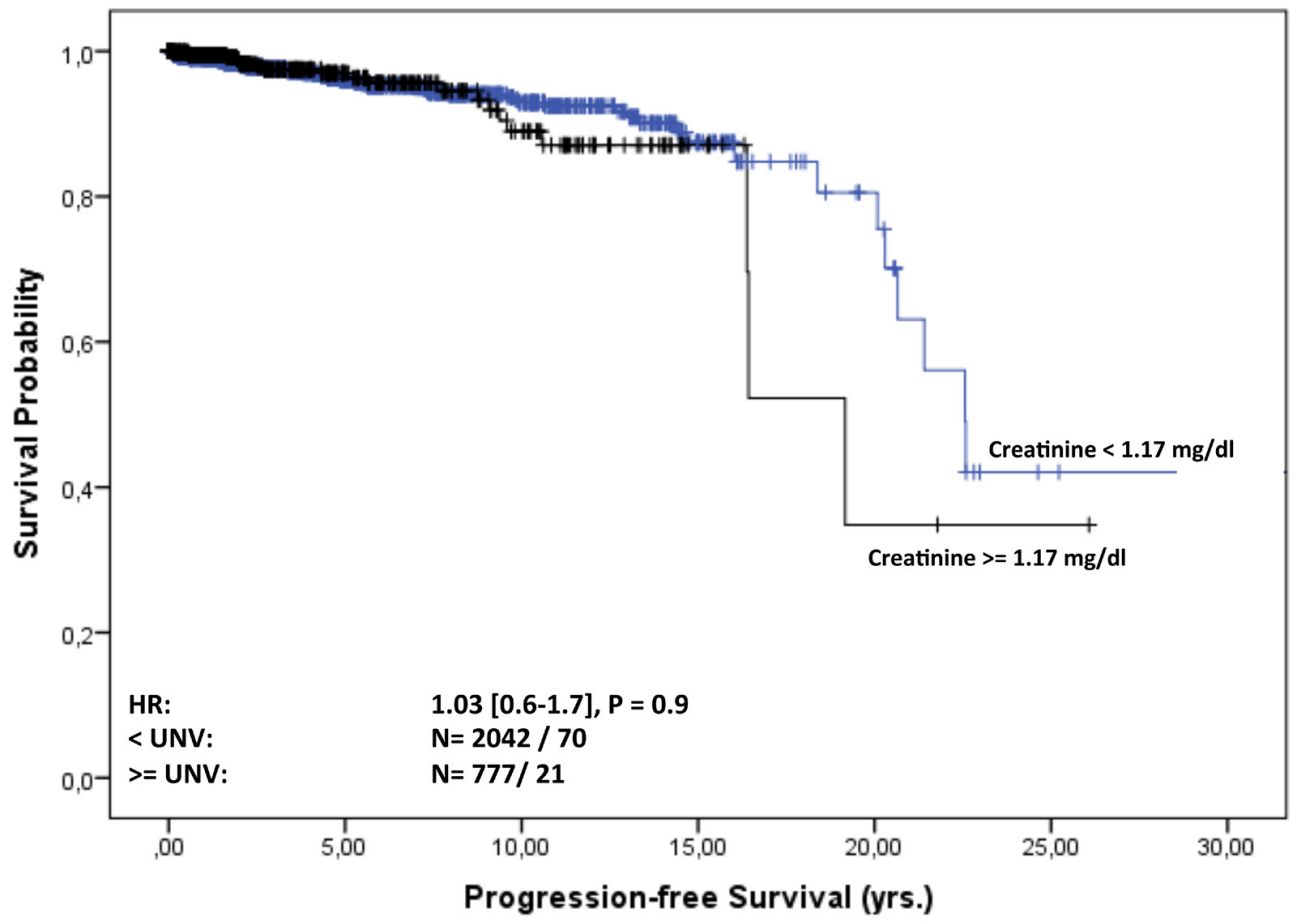
Progression-free survival of MGUS patients from diagnosis stratified by normal and elevated creatinine level at baseline Progression-free survival of MGUS patients in years from diagnosis stratified by creatinine level at baseline. The hazard ratio (HR, 95% CI) was calculated with a Cox regression model adjusted for sex and age.

**Figure 5:**
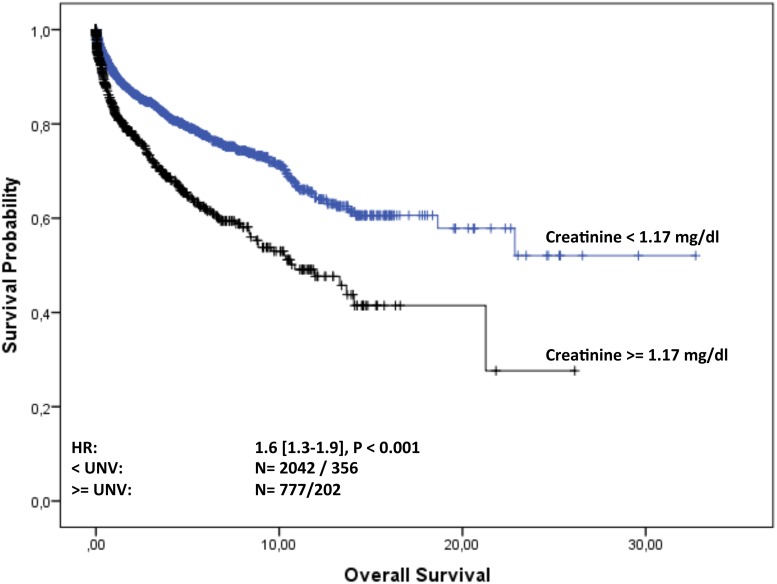
Overall survival of MGUS patients from diagnosis stratified by normal and elevated creatinine level at baseline Overall survival of MGUS patients in years from diagnosis stratified by creatinine level at baseline. The hazard ratio (HR, 95% CI) was calculated with a Cox regression model adjusted for sex and age.

Overall follow-up time for the 2935 patients was 11,050 person-years (median 23 months [IQR: 4-70] per patient) and maximum observation time was 32.8 years. Median time at risk was 23 months [IQR: 4-69] for MGUS patients and 61 months [IQR: 17-125] for MGRS patients. Of the 2891 MGUS patients 566 (20%) and of the 44 MGRS patients eight (18%) died during follow-up. Median survival in the cohort was 23 years. Of the MGUS patients 75% were alive after 5.4 years, whereas 75% of the MGRS patients survived 9.5 years. For MGUS patients we estimated a mortality rate of 52 [95% CI 48-56] per 1000 patient-years, whereas for MGRS patients the rate was 29 [14-58] per 1000 patient-years. The estimated risk for death within the first year after diagnosis was 10% [9-11] in the MGUS group and 2.5% [0.3-16] in the MGRS group. Kaplan-Meier survival curves for MGUS and MGRS did not differ significantly (P_logrank_=0.2; Figure 2). Only 92 (3%) of the 2891 MGUS patients and notably eight (18%) of the 44 MGRS patients progressed during time of observation. Of the MGUS patients 75% did not show progression for the first 19 years, whereas this time-span shrunk to 11 years for MGRS patients. Median time to progression was 23 years for MGUS and 18.8 years for MGRS patients. The corresponding progression rate for MGUS patients was 8.8 [7.2-10.7] per 1000 patient-years and 30.6 [15.3–61] in the MGRS group. The risk for progression within the first year after diagnosis was 1% [0.6-1.4] in the MGUS group and 10% [4-29] among MGRS patients Kaplan-Meier analysis revealed a significant difference in cumulative progression between MGUS and MGRS patients (P_logrank_<0.001), and MGRS patients showed a significantly higher risk for progression (HR 3.3 [1.5-7.4], Figure 3). Corresponding results were observed when MGUS patients with elevated creatinine levels at baseline (>= 1.17 mg/dl) were excluded in a sensitivity analysis (HR 4.6 [2.0 – 10.1], p<0.001). Concerning progression-free-survival, no significant differences between patients with vs. without elevated creatinine levels was observed within the MGUS stratum (Figure 4), whereas overall survival was worse for MGUS patients with elevated creatinine levels (HR 1.6 [1.3-1.9], P_logrank_ <0.001; Figure 5).

### Risk factors for disease progression

**Figure 6:**
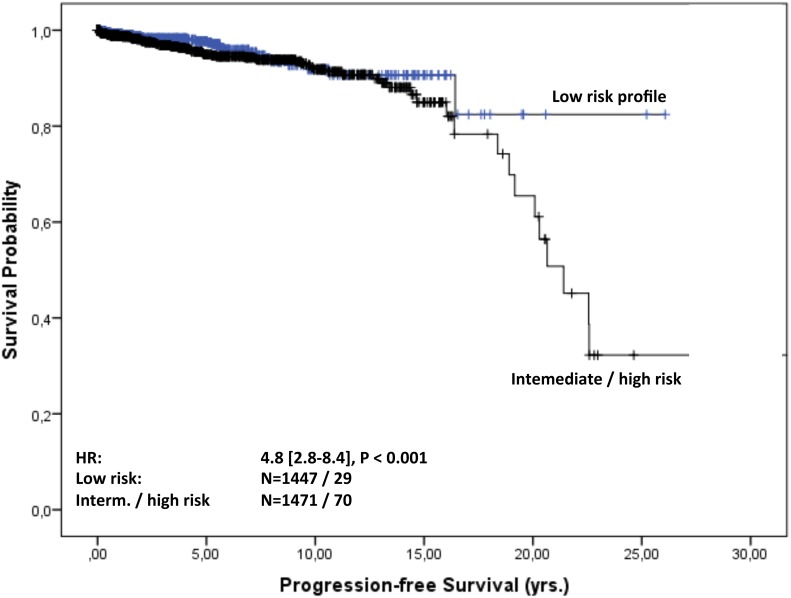
Progression-free survival of risk strata Progression-free survival in years from MGUS diagnosis stratified by the risk group profile based on the M protein concentration, Ig type and FLC ratio.

MGUS and MGRS patients were additionally evaluated for clinical risk factors for disease progression. Compared to the IgG isotype group as a reference, patients with light-chain only faced an almost 4-fold risk for progression (HR 3.9 [1.6-9.7], P=0.003). The hazard for IgA subtypes yielded 2.3 [1.3-4.1] as compared to the IgG isotype (P=0.003). This result was confirmed using an established risk stratification model based on M protein concentration, Ig type and level of FLC ratio in comparison to the low-risk reference group (all factors normal, n= 1447). Patients with low intermediate risk (one factor positive, n= 1143) showed a 2-fold risk (OR= 1.95 [1.2-3.2], P=0.007) for malignant progression, whereas the aggregated group with high or intermediately high risk (at least two positive factors, n = 328) faced an almost 5-fold risk (OR = 4.8 [2.8-8.4], P<0.001, Figure 6). Progressed MGRS patients (n=8) showed a significantly higher frequency of the IgG isotype (n=5) and light-chain only immunoglobulin (n=3) than did the 92 progressed MGUS patients (IgG: n=52; light-chain only: n=3). Abnormal serum creatinine levels at diagnosis were not associated with malignant hematologic progression in the overall cohort. In contrast, MGRS patients with reduced renal function during follow-up (doubling of serum creatinine, or renal replacement therapy) showed a 2.6-fold risk for hematologic progression (OR=2.6 [CI: 0.3 – 24]).

## DISCUSSION

In this sizeable observational study, outcomes in 2935 patients with MGUS and MGRS were analysed regarding clinical features and risk factors for haematological and renal progression. A nephropathy secondary to monoclonal gammopathy of renal significance (MGRS) is often underestimated and a rare condition. This term was first used in 2012 by Leung et al. [8]. Except for a few case reports the current literature does not reveal large studies of MGRS patients [12-15]. Sethi and colleagues presented a recent study with 68 patients with membranoproliferative glomerulonephritis (MPGN) secondary to monoclonal gammopathy [16]. The special feature of our study is a very large number of patients and a long follow-up time of over 20 years.

In our study laboratory features (immunoglobulins, Ig light chains, renal dysfunction, elevated creatinine, urea, and phosphate levels, progression to various haematological diseases) differed between MGUS and MGRS. We found a significantly higher incidence of elevated creatinine, urea, phosphate levels and IgG kappa in MGRS. An association between the higher prevalence of IgG immunoglobulins in the MGRS cohort including chronic kidney disease has already been described [11, 17].

Kyle and coworkers described the annual risk for progression to MM in patients with MGUS as being about 1% [5]. Patients with light-chain MGUS have a 0.3% risk for progression to MM [18]. Our results are in line with the findings made by Kyle and coworkers, but differ between the two strata (progression rate 1% /year in the MGUS cohort vs. 10% /year in the MGRS cohort). A recent study by Ciocchini and colleagues described an estimated prevalence of MGRS of about 0.32% and 0.53%, depending on the population age [19]. In our cohort, 44 (1.5%) of the 2935 MGUS patients presented with MGRS. Kaplan-Meier analysis revealed a significant difference in cumulative progression between MGUS and MGRS patients (P_logrank_<0.001). Patients affected by MGRS, progressed significantly more often to MM than did MGUS patients. Several risk factors are associated with progression to MM, like immunoglobulin isotype, the M protein concentration and the free light-chain ratio [5, 20, 21]. Important for MGUS patients is regular follow-up that may be associated with outcome in MM [22]. International guidelines recommend annual monitoring for high-risk MGUS patients and follow-up in low-risk MGUS patients [20]. Monoclonal gammopathy is associated with a variety of kidney diseases [19]. Monoclonal gammopathy-associated renal diseases differ in pathogenesis, clinical manifestation, several kidney biopsy findings, disease progression, outcome, and therapy strategies [23]. This complex term describes patients with renal impairment secondary to monoclonal immunoglobulin secreted by premalignant or malignant clones [23]. In our MGRS cohort elevated creatinine levels at follow-up were found to be a risk factor for disease progression to haematological disorders. Hitherto there exist only few case series but no prospective data about this observation. Nevertheless, it is important knowledge and should be taken into account as a risk factor for disease progression in future, especially for MGRS patients. As MGRS is related to high morbidity because of the renal and systematic lesion caused by monoclonal immunoglobulin, it is of utmost importance to recognize this circumstance as early as possible because adequate therapy can significantly improve patient survival [11]. For patients who are suspected of having MGRS it is important to diagnose the type of monoclonal gammopathy, the pathogenic cell clone and the type of nephropathy [11]. The best way to diagnose the deposits of monoclonal immunoglobulin in the kidney is to perform a kidney biopsy. To better characterize the specific lesion immunofluorescence and electron microscopy can be used [11]. The poor prognosis and the higher risk for progression to a haematological malignancy for MGRS patients in our cohort as compared to MGUS patients could be associated with the failure to undergo regular follow-ups conducted by appropriate specialists and inconsistent, delayed, or no treatment received by these patients. Of note, 72% of the MGRS patients in our cohort develop deterioration of renal function and anti-myeloma substances could favourably influence renal prognosis. Hence, regular haematological and renal follow-up is recommended [24]. While there are no standardized guidelines for the treatment of MGUS patients, MGRS diagnosis makes therapy necessary [10]. MGRS treatment should focus on rapid suppression of the toxic monoclonal immunoglobulin that damages the renal system [10]. The therapeutic option should focus on the plasma cell clone with appropriate chemotherapy. Making the right choice of chemotherapeutic agent requires that renal toxicity and renal metabolism be considered [10]. When treating the renal lesion, MGRS patients should be continuously monitored by a nephrologist, including for proteinuria, hypertension and serum creatinine [25].

In conclusion, in our large observational study we found differences in clinical features and risk factors for disease progression in MGUS and MGRS patients. Nevertheless, a small proportion (1.5%) of patients had nephropathy secondary to monoclonal gammopathy of renal significance. MGRS is a rare disease, but a complex problem in clinical practice. We recommend that MGUS patients be carefully monitored and referred to a specialized nephrological and haematological centre if creatinine levels are elevated or renal symptoms occur. Even more, in MGUS patients with increased creatinine levels secondary to paraproteinemia a renal biopsy should be performed to ensure the correct diagnosis. The main objective for MGRS patients with high risk for progression to MM or other related diseases is to detect early progression to MM and prevent complications. MGUS patients should be considered for risk- and response-stratified therapy monitoring in terms of renal manifestations in order to improve their quality of life. Furthermore, the lack of standardized therapies based on prospective studies means individualized treatment options should be offered in patients with renal impairment, depending on renal biopsy findings. A well-functioning cooperation between nephrologists and haematologists is absolutely necessary for an individualized diagnostic and therapeutic approach to improve outcome in these rare and complex patients. The data presented here could certainly be useful in designing future prospective studies.

## MATERIALS AND METHODS

### Patients

Between January 2000 and August 2016, a total of 2935 adult patients with MGUS were identified in our local database. The patients were either assessed on serological basis (monoclonal protein in the serum of <3 g/dl) or fulfilled the criteria for MGUS according to the International Myeloma Working Group (IMWG) criteria [12]. Sampling of patient data was approved by the local institutional ethics committee (vote number: AN2015-0193 352/4.13) and was conducted in accordance with the principles of the Declaration of Helsinki. The observation period in some patients lasted over 20 years since the initial diagnosis was made before 2000. MGUS and MGRS patients were compared and analysed for disease progression. MGRS patients were diagnosed by a consultant nephrologist. In total, 129 patients were identified who either had serum creatinine above normal or proteinuria greater or equal to 150 mg/g or were dependent on renal replacement therapy. Screening of physician letters enabled 44 patients to be diagnosed as suffering from MGRS. In particular, 25 of the 44 patients had a renal-biopsy-proven renal disease that was attributed to MGUS (see Table 2). In five additional patients renal AL amyloidosis was histopathologically diagnosed in another non-renal solid organ (n=1 bone-marrow, n=3 heart, n=1 stomach/duodenum) [26-29]. Hence, the diagnosis of MGRS was biopsy-proven in 73% of patients. Of the patients without (non-) renal biopsy (27%), physician letters revealed five patients with unexplained nephrotic syndrome, of whom three had MGUS and two had massive free light chain proteinuria and were therefore assigned to MGRS. In seven cases of unexplained chronic proteinuric kidney disease (median serum creatinine 1.72 mg/dl [95%CI: 1.39 - 2.07]; median proteinuria 1457 mg/g [95%CI: 1058 - 2078]) that had no underlying co-morbidity (e.g. diabetes, hypertension) other than MGUS and had been diagnosed as MGRS by a nephrologist, the diagnosis of MGRS was confirmed by an independent consultant nephrologist.

### Statistical analysis

Descriptive statistics were used to summarize the sample, laboratory measures and disease outcomes stratified by MGUS vs. by MGRS. All event summaries refer to the first hematologic malignancy (progression-free survival) and death (overall survival). Chi-squared test, MW-U test and survival analysis (Kaplan-Meier curves, log-rank test) were used to test for differences between MGUS and MGRS patients. To obtain crude estimates of odds ratios (OR) a logistic regression model was used. We calculated crude incidence rates as the number of events divided by the total number of person-years at risk following MGUS diagnosis, and 95% confidence intervals (CIs) were based on a Poisson distribution. We estimated unadjusted cumulative 1y-risk for mortality and progression, defined as the probability of the event within the first year following MGUS diagnosis. Using Cox proportional hazards models, we examined the hazard ratio associations (HRs) between progression for MGUS / MGRS and IG subtypes adjusted for sex, age (continuous), and creatinine at baseline. Time scale for calculation of the Cox proportional hazards models was months from MGUS diagnosis. For visualization, the time scale was changed to years. The proportional hazards assumption was tested by inspecting Kaplan-Meier curves and using Schoenfeld residuals. All tests for statistical significance were two-sided. P values less than 0.05 were considered statistically significant, and for point estimators we provide 95% confidence intervals (CI). Statistical evaluation was performed using SPSS statistical software (version 20.0; SPSS Inc., Chicago, IL, USA) and Stata (version 14, StataCorp LLC, College Station, TX, USA).

## References

[1] Kyle RA (1978). Monoclonal gammopathy of undetermined significance. Natural history in 241 cases. Am J Med.

[2] Kyle RA, Therneau TM, Rajkumar SV, Larson DR, Plevak MF, Offord JR, Dispenzieri A, Katzmann JA, Melton LJ (2006). Prevalence of monoclonal gammopathy of undetermined significance. N Engl J Med.

[3] Munshi NC (2007). Monoclonal gammopathy of undetermined significance: genetic vs environmental etiologies. Mayo Clin Proc.

[4] Merlini G, Stone MJ (2006). Dangerous small B-cell clones. Blood.

[5] Kyle RA, Durie BG, Rajkumar SV, Landgren O, Blade J, Merlini G, Kroger N, Einsele H, Vesole DH, Dimopoulos M, San Miguel J, Avet-Loiseau H, Hajek R (2010). Monoclonal gammopathy of undetermined significance (MGUS) and smoldering (asymptomatic) multiple myeloma: IMWG consensus perspectives risk factors for progression and guidelines for monitoring and management. Leukemia.

[6] Dimopoulos MA, Terpos E, Chanan-Khan A, Leung N, Ludwig H, Jagannath S, Niesvizky R, Giralt S, Fermand JP, Blade J, Comenzo RL, Sezer O, Palumbo A (2010). Renal impairment in patients with multiple myeloma: a consensus statement on behalf of the International Myeloma Working Group. J Clin Oncol.

[7] Nasr SH, Said SM, Valeri AM, Sethi S, Fidler ME, Cornell LD, Gertz MA, Dispenzieri A, Buadi FK, Vrana JA, Theis JD, Dogan A, Leung N (2013). The diagnosis and characteristics of renal heavy-chain and heavy/light-chain amyloidosis and their comparison with renal light-chain amyloidosis. Kidney Int.

[8] Leung N, Bridoux F, Hutchison CA, Nasr SH, Cockwell P, Fermand JP, Dispenzieri A, Song KW, Kyle RA (2012). Group IKaMGR. Monoclonal gammopathy of renal significance: when MGUS is no longer undetermined or insignificant. Blood.

[9] Solomon A, Weiss DT, Kattine AA (1991). Nephrotoxic potential of Bence Jones proteins. N Engl J Med.

[10] Fermand JP, Bridoux F, Kyle RA, Kastritis E, Weiss BM, Cook MA, Drayson MT, Dispenzieri A, Leung N (2013). Group IKaMGR. How I treat monoclonal gammopathy of renal significance (MGRS). Blood.

[11] Bridoux F, Leung N, Hutchison CA, Touchard G, Sethi S, Fermand JP, Picken MM, Herrera GA, Kastritis E, Merlini G, Roussel M, Fervenza FC, Dispenzieri A (2015). Diagnosis of monoclonal gammopathy of renal significance. Kidney Int.

[12] Vankalakunti M, Bonu R, Shetty S, Siddini V, Babu K, Ballal SH (2014). Crystalloid glomerulopathy in monoclonal gammopathy of renal significance (MGRS). Clin Kidney J.

[13] Fatima R, Jha R, Gowrishankar S, Narayen G, Rao BS (2014). Proliferative glomerulonephritis associated with monoclonal immune deposits: a case report and review of literature. Indian J Nephrol.

[14] Nasr SH, Satoskar A, Markowitz GS, Valeri AM, Appel GB, Stokes MB, Nadasdy T, D’Agati VD (2009). Proliferative glomerulonephritis with monoclonal IgG deposits. J Am Soc Nephrol.

[15] Sethi S, Sukov WR, Zhang Y, Fervenza FC, Lager DJ, Miller DV, Cornell LD, Krishnan SG, Smith RJ (2010). Dense deposit disease associated with monoclonal gammopathy of undetermined significance. Am J Kidney Dis.

[16] Sethi S, Zand L, Leung N, Smith RJ, Jevremonic D, Herrmann SS, Fervenza FC (2010). Membranoproliferative glomerulonephritis secondary to monoclonal gammopathy. Clin J Am Soc Nephrol.

[17] Kapoulas S, Raptis V, Papaioannou M (2015). New aspects on the pathogenesis of renal disorders related to monoclonal gammopathies. Nephrol Ther.

[18] Dispenzieri A, Katzmann JA, Kyle RA, Larson DR, Melton LJ, Colby CL, Therneau TM, Clark R, Kumar SK, Bradwell A, Fonseca R, Jelinek DF, Rajkumar SV (2010). Prevalence and risk of progression of light-chain monoclonal gammopathy of undetermined significance: a retrospective population-based cohort study. Lancet.

[19] Ciocchini M, Arbelbide J, Musso CG (2017). Monoclonal gammopathy of renal significance (MGRS): the characteristics and significance of a new meta-entity. Int Urol Nephrol.

[20] www.onkopedia.com/de/onkopedia/guidelines/monoklonale-gammopathie-unklarer-signifikanz-mgus/@@view/html/index.html

[21] Turesson I, Kovalchik SA, Pfeiffer RM, Kristinsson SY, Goldin LR, Drayson MT, Landgren O (2014). Monoclonal gammopathy of undetermined significance and risk of lymphoid and myeloid malignancies: 728 cases followed up to 30 years in Sweden. Blood.

[22] Sigurdardottir EE, Turesson I, Lund SH, Lindqvist EK, Mailankody S, Korde N, Bjorkholm M, Landgren O, Kristinsson SY (2015). The role of diagnosis and clinical follow-up of monoclonal gammopathy of undetermined significance on survival in multiple myeloma. JAMA Oncol.

[23] Sethi S, Fervenza FC, Rajkumar SV (2016). Spectrum of manifestations of monoclonal gammopathy-associated renal lesions. Curr Opin Nephrol Hypertens.

[24] Vignon M, Cohen C, Faguer S, Noel LH, Guilbeau C, Rabant M, Higgins S, Hummel A, Hertig A, Francois H, Lequintrec M, Vilaine E, Knebelmann B (2017). The clinicopathologic characteristics of kidney diseases related to monotypic IgA deposits. Kidney Int.

[25] Correia SO, Santos S, Malheiro J, Cabrita A, Martins S, Santos J (2017). Monoclonal gammopathy of renal significance: diagnostic workup. World J Nephrol.

[26] Kyle RA, Greipp PR, Amyloidosis AL (1983). Clinical and laboratory features in 229 cases. Mayo Clin Proc.

[27] Duston MA, Skinner M, Shirahama T, Cohen AS (1987). Diagnosis of amyloidosis by abdominal fat aspiration. Analysis of four years’ experience. Am J Med.

[28] Duston MA, Skinner M, Meenan RF, Cohen AS (1989). Sensitivity, specificity and predictive value of abdominal fat aspiration for the diagnosis of amyloidosis. Arthritis Rheum.

[29] Sungur C, Sungur A, Ruacan S, Arik N, Yasavul U, Turgan C, Caglar S (1993). Diagnostic value of bone marrow biopsy in patients with renal disease secondary to familial Mediterranean fever. Kidney Int.

